# Glucose-6-phosphate dehydrogenase deficiency among malaria patients of Honduras: a descriptive study of archival blood samples

**DOI:** 10.1186/s12936-015-0823-z

**Published:** 2015-08-07

**Authors:** Miguel Á Zúñiga, Rosa E Mejía, Ana L Sánchez, Wilfredo H Sosa-Ochoa, Gustavo A Fontecha

**Affiliations:** Microbiology Research Institute, National Autonomous University of Honduras, UNAH, Boulevard Suyapa, J1 Building, 4th Fl. Tegucigalpa, Francisco Morazán, Honduras; Pan American Health Organization, Tegucigalpa, Honduras; Department Health Sciences, Brock University, 500 Glenridge Avenue, St Catharines, Ontario, ON L2S 3A1 Canada

**Keywords:** Glucose-6-phosphate dehydrogenase deficiency, Malaria, Honduras, *Plasmodium vivax*, Primaquine

## Abstract

**Background:**

The frequency of deficient variants of glucose-6-phosphate dehydrogenase (G6PDd) is particularly high in areas where malaria is endemic. The administration of antirelapse drugs, such as primaquine, has the potential to trigger an oxidative event in G6PD-deficient individuals. According to Honduras´ national scheme, malaria treatment requires the administration of chloroquine and primaquine for both *Plasmodium vivax* and *Plasmodium falciparum* infections. The present study aimed at investigating for the first time in Honduras the frequency of the two most common G6PDd variants.

**Methods:**

This was a descriptive study utilizing 398 archival DNA samples of patients that had been diagnosed with malaria due to *P. vivax*, *P. falciparum,* or both. The most common allelic variants of G6PD: G6PD A+^376G^ and G6PD A−^376G/202A^ were assessed by two molecular methods (PCR–RFLP and a commercial kit).

**Results:**

The overall frequency of G6PD deficient genotypes was 16.08%. The frequency of the “African” genotype A− (Class III) was 11.9% (4.1% A− hemizygous males; 1.5% homozygous A− females; and 6.3% heterozygous A− females). A high frequency of G6PDd alleles was observed in samples from malaria patients residing in endemic regions of Northern Honduras. One case of Santamaria mutation (376G/542T) was detected.

**Conclusions:**

Compared to other studies in the Americas, as well as to data from predictive models, the present study identified a higher-than expected frequency of genotype A− in Honduras. Considering that the national standard of malaria treatment in the country includes primaquine, further research is necessary to ascertain the risk of PQ-triggered haemolytic reactions in sectors of the population more likely to carry G6PD mutations. Additionally, consideration should be given to utilizing point of care technologies to detect this genetic disorder prior administration of 8-aminoquinoline drugs, either primaquine or any new drug available in the near future.

**Electronic supplementary material:**

The online version of this article (doi:10.1186/s12936-015-0823-z) contains supplementary material, which is available to authorized users.

## Background

Honduras is a malaria-endemic country where the two circulating parasite species are *Plasmodium vivax* and *Plasmodium falciparum*, with *P. vivax* being responsible for 79% of symptomatic cases, which represent more than 4,000 cases a year [1]. Honduras has a population of approximately 8.7 million, of which 93% is considered mestizo (persons of mixed Amerindian and European ancestry). Indigenous peoples comprise seven minority ethnic groups, making up for less than half a million people. The existing ethnic groups of African descent represent about 5% of the population [2] and are concentrated on the Atlantic coast of Northern Honduras, where the incidence of malarial infections is higher.

Treatment of *P. vivax* malaria includes the 8-aminoquinoline primaquine (PQ), to prevent relapses due to dormant liver stages [3]. National guidelines in Honduras follow the World Health Organization (WHO) recommendation of 0.25 mg/kg over 14 days for use as a radical cure; i.e., to prevent *P. vivax* relapse [4]. However, in some countries this treatment is contraindicated in patients with severe deficiency of glucose-6-phosphate dehydrogenase (G6PD), as PQ could trigger acute haemolysis and/or removal of red blood cells (RBC) by the spleen [5, 6].

The geographical distribution of this genetic disorder extends through Africa, Asia, Southeast Asia and Latin America and overlaps with that of malaria [7]. Recent research suggests that as malaria elimination efforts intensify, identifying G6PD-deficient (G6PDd) individuals prior to treatment is essential to protect patients from potential haemolytic reactions [8]. G6PD is an essential enzyme present in all cells and is involved in producing the necessary NADPH required for protecting cells against oxidative stress. While G6PD deficiency can be compensated by alternate pathways in other cells, NADPH is the only mechanism to counter balance oxidative stress in the erythrocyte [9, 10].

The G6PD locus is sex-linked, and the deficiency of this enzyme is one of the most prevalent polymorphisms causing hereditary enzymopathies in humans, particularly among males who are hemizygous for this trait [10–12]. Numerous mutations (186 or more) have been identified in the G6DP gene [8, 13], resulting in >400 biochemical variants [14–16].

Due to the large number of variants and differences in enzymatic activity, creating a classification system with corresponding public health and clinical significance has been challenging. A recent proposal, as reviewed by Howes et al. [17] suggests a simplified classification including only three types of variants, as follows. Type 1: rare, with <10% of enzyme activity, clinically severe and chronic; Type 2: more common, with residual enzyme activity <1–50%, clinically asymptomatic until triggered by an external event; and Type 3: those with more than 50% of enzyme activity and, therefore, without clinical significance [17, 18]. This classification is not widely used. Instead, the WHO recommends utilizing Yoshida’s taxonomy, consisting of five classes depending on enzymatic activity and clinical manifestations [19, 20]. The most severe deficiency is categorized as Class I, with variants that albeit rare, are associated with chronic haemolytic anaemia. Higher-frequency variants belong to classes II and III, and are associated with some degree of protection against malaria. Class IV and V are considered or normal or with increased activity, respectively [19]. Among the more prevalent variants, the so-called “African” genotype A−^202A^ with 12% of enzymatic activity is classified as class III, and individuals with such variant experience clinical manifestations after specific triggers [17]. In turn, genotype A+ gives rise to very-mild deficient or normal variants and is included in class IV [21–23].

It is widely recognized that deficient variants of G6PD are more common in African, Mediterranean, and Asian populations [24]. However, research evidence is scant from some areas of the world. Recently, Howes et al. set out to characterize the spatial distribution of G6PD deficiency variants in areas where malaria is endemic, and their findings underscore the scarcity of data from the Americas [17]. Similar findings are reported by Monteiro et al. [25]. In the Central American region, only Panama [26] and Costa Rica [27–30] register publications reporting the prevalence of G6PDd. Paradoxically, similar data from Honduras, the country with the highest burden of malaria in the region [1, 31], is non-existent—no local publications or reports in the grey literature could be found for the present analysis. No information on the relevance of primaquine-associated haemolysis in Honduras is available either.

The present study aimed at investigating the frequency of the most common G6PDd variants in a subset of Honduran malaria patients. This preliminary information will be useful to determine the need for larger population-based prevalence studies to assess the potential risk of haemolytic anaemia due to PQ administration in populations living in malaria-endemic areas.

## Methods

### Study area, population and design

**Fig. 1:**
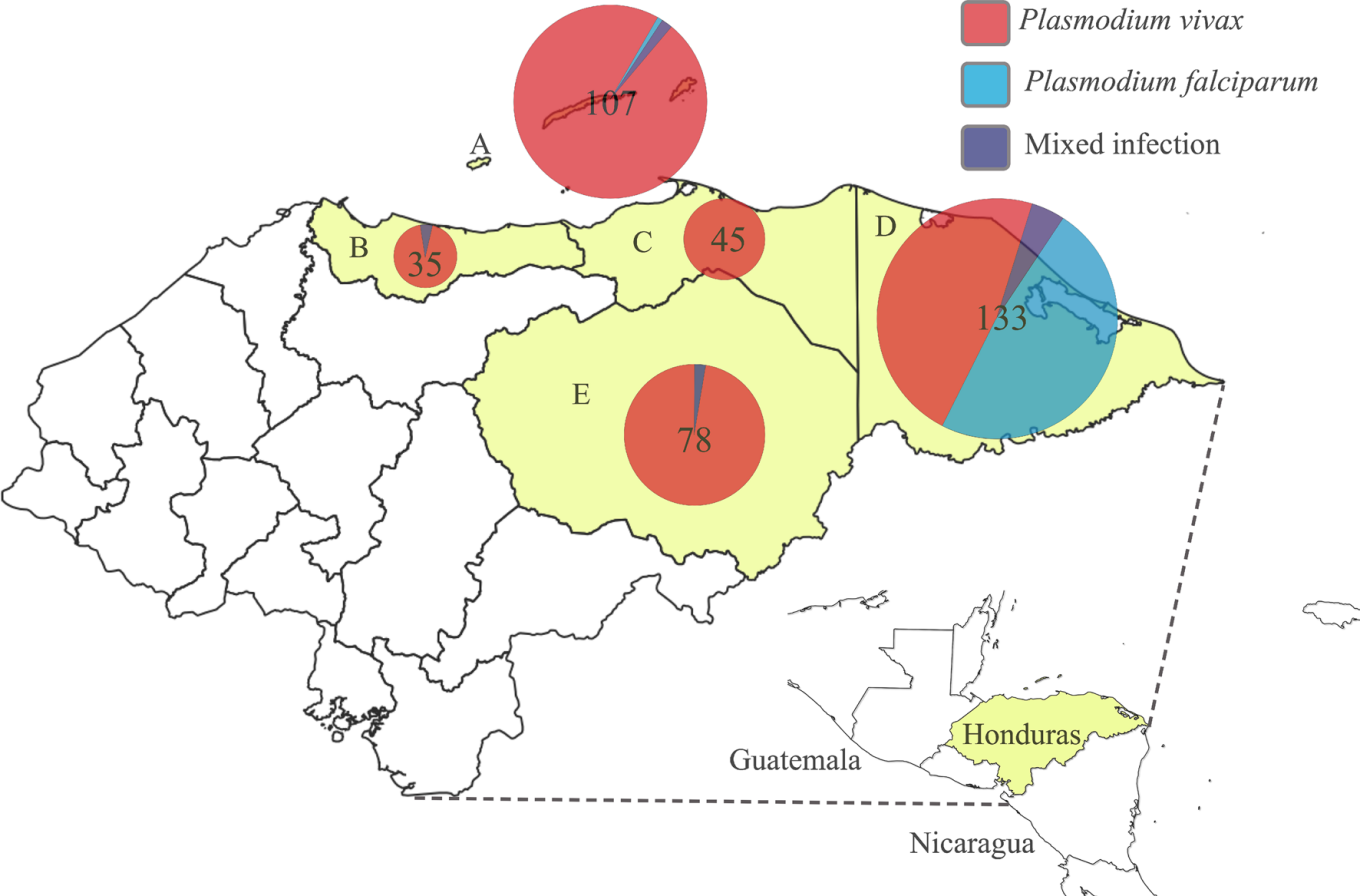
Map of Honduras and number of samples collected in five departments showing the parasite species causing the malaria infection.

This descriptive study included a total of 400 blood samples from the same number of Honduran malaria patients and collected onto Whatman FTA^®^ cards (GE Healthcare). Patients had been routinely assessed for malaria by the Honduran Ministry of Health laboratory network both by standard microscopy and subsequent confirmation by molecular analysis. Cards had been stored in the molecular biology laboratory at the National Autonomous University of Honduras (UNAH), and archived by malaria species diagnosis and patient’s provenance, but devoid of patients’ personal information. Other than malaria infection, no other clinical information (e.g. health status, haemolytic anaemia, etc.) was associated with the samples. According to the place of origin, samples were from 22 municipalities located in five malaria-endemic departments (second-level political jurisdictions) of Honduras: Atlántida (n = 35), Colón (n = 45), Gracias a Dios (n = 133), Islas de la Bahía (n = 107), and Olancho (n = 78) (Fig. 1).

### Ethical clearance

The study made secondary use of biological specimens originally collected for malaria diagnosis as per standard of care in Honduras. However, as mentioned earlier, blood samples had been anonymized. That is, blood samples had been irrevocably stripped of direct identifiers, so future re-identification of individuals and linkage to the G6PD findings was not possible. Scientific approval and ethical clearance was obtained from the Ethics Review Committee of the Infectious and Zoonotic Diseases Masters Program at UNAH (CEI-MEIZ 02-2014; 5/19/2014).

### Malaria microscopic and molecular diagnosis

As mentioned earlier, samples belonged to malaria-positive patients previously determined by both microscopy and molecular biology. Briefly, experienced microscopists at the Ministry of Health laboratories throughout the country carried out malaria diagnosis after Giemsa-staining of thick and thin blood smears. Diagnosis confirmation was done at UNAH as follows. Genomic DNA was extracted from *Plasmodium*-positive filter paper blood spots using a Chelex-based method [32]. The detection of *Plasmodium* sp. DNA was performed using an 18S rRNA nested PCR approach, according to Singh et al. [33]. Based on both diagnostic methods (microscopy and molecular), 322 (80.90%) samples were positive for *P. vivax,* 66 (16.58%) for *P. falciparum,* and 10 (2.51%) for both species (mixed infections) (Table 1).

**Table 1:** Number of samples collected by sex and parasite species causing the infection

Department	n	Sex	Total	*P. vivax*	*P. falciparum*	Mixed infection
			n (%)	n (%)	n (%)	n (%)
Atlántida	35	M	16 (4.02)	15 (3.77)	0	1 (0.25)
		F	19 (4.77)	18 (4.52)	0	1 (0.25)
Colón	45	M	19 (4.77)	19 (4.77)	0	0
		F	26 (6.53)	26 (6.53)	0	0
Gracias a Dios	133	M	59 (14.82)	29 (7.29)	28 (7.04)	2 (0.50)
		F	74 (18.59)	35 (8.79)	35 (8.79)	4 (1.01)
Islas de la Bahía	107	M	45 (11.31)	42 (10.55)	1 (0.25)	2 (0.50)
		F	62 (15.58)	62 (15.58)	0	0
Olancho	78	M	45 (11.31)	44 (11.06)	1 (0.25)	0
		F	33 (8.29)	32 (8.04)	1 (0.25)	0
Total	398	M	184 (46.23)	149 (37.44)	30 (7.54)	5 (1.26)
		F	214 (53.77)	173 (43.47)	36 (9.05)	5 (1.26)
			398 (100)	322 (80.90)	66 (16.58)	10 (0.02)

Since samples were stripped from personal identifiers, it was necessary to determine patients’ sex through a molecular approach. Two regions of the human sex chromosomes were amplified simultaneously, as described by Settin et al. [34]. Briefly, two sets of primers were used in a multiplex PCR. SRYF: 5´-CAT GAA CGC ATT CAT CGT GTG GTC-3´ and SRYR: 5´-CTG CGG GAA GCA AAC TGC AAT TCT T-3´ for the Y chromosome, and ALTF: 5´-CCC TGA TGA AGA ACT TGT ATC TC-3´/ALTR: 5´-GAA ATT ACA CAC ATA GGT GGC ACT-3´ for the X chromosome, with amplicon sizes of 254, and 300 bp, respectively.

PCR reactions were performed in a 50-μL reaction volume containing Taq polymerase master mix (Promega Corp.), 4  pmol of each primer, and 40  ng of DNA. PCR conditions were as follow: 2 min at 94°C and 35 cycles of 94°C for 30 s, 66°C for 30 s and 72°C for 30 s, and a final extension of 15 min at 72°C. PCR products were visualized on 2% agarose gels.

### PCR–RFLP for G6PD

Two DNA fragments of the G6PD gene, corresponding to exons IV (codon 68, cDNA nucleotide substitution 202) and V (codon 126, cDNA nucleotide substitution 376) were PCR amplified independently according to protocols previously described [22, 35], with some modifications. Briefly, 20 ng of DNA were added to a final volume of 50 μL in a reaction mixture containing 2X Taq polymerase Master Mix (Promega Corp.) and 0.4 μM of each primer. Primer sequences are shown in Table 2. Reaction conditions were as follow: For the region including the codon 202: 94°C/10 min, 39 cycles of 94°C/2 min, 66°C/1 min, and 72°C/2 min. For the region including the codon 376: 94°C/10 min, 35 cycles of 94°C/2 min, 68°C/1 min, and 72°C/2 min. A final extension step of 10 min was included for all reactions. PCR products of 898 and 585 bp, respectively were detected in agarose gels.

**Table 2:** Primer sequences, exons targeted, and restriction enzymes used in the PCR-RFLP assay to detect two G6PD mutations

Variant	Sequence (5´–3´)	Exon	Restriction enzyme	References
A− (202 G>A, 376 A>G)	GTGGCTGTTCCGGGATGGCCTTCTG AGGGCAACGGCAAGCCTTAC	IV, V	NlaIII	[22, 35]
A+ (376 A>G)	CTGCGTTTTCTCCGCCAATC AGGGCAACGGCAAGCCTTAC	V	FokI	[22, 35]

G6PD genotyping was carried out by RFLP of the PCR products in order to detect two of the most common African variants, G6PD A+ and G6PD A− [36]. The 376 A → G mutation leading to the G6PD A+ variant was detected through digestion with FokI. All samples showing a G6PD A+ pattern were further analyzed for the 202 G → A mutation, characteristic of the G6PD A− variant. This genotype was detected by digestion with NlaIII as previously described [22]. All digestions were performed at 37°C according to the manufacturer’s instructions (New England Biolabs) in a final reaction volume of 20 μL. FokI digestion was incubated for 60 min and NlaIII for 50 min. A 2.5% agarose gel was required to separate properly the restriction fragments.

The expected pattern of digestion for all genotypes (Table 3) was confirmed in silico using the Geneious 7.1.7 software [37] with wild type and mutant sequences obtained from the OMIM database [38].

**Table 3:** Restriction patterns of normal and mutant individuals by PCR–RFLP

G6PD variant	Fragment size (bp)			
	Restriction patterns			
	PCR product	Wild type	Mutant	References
A− (202 G>A, 376 A>G)	898	423, 184, 169, 106, 32	423, 184, 127, 106, 46	[22, 35]
A+ (376 A>G)	585	402, 187	289, 187, 117	[22, 35]

### Confirmation of G6PD variants (Multiplex PCR and sequencing)

In addition to the PCR–RFLP analysis, all samples showing a G6PD deficient genotype were screened using DiaPlexC™ G6PD Genotyping Kit, African type (SolGent, Co., Ltd.), which enables to detect 6 different G6PD variants by a one-step multi-allelic specific PCR. The six variants amplify PCR products of different sizes, as follows: G6PD A− (202) (376A → G, 103 bp; 202G → A, 157 bp); G6PD A+ (376A → G, 103 bp); G6PD Santamaria (376A → G, 103 bp; 542A → T 241 pb); G6PD A− (680) (376A → G, 103 bp; 680G → T, 388 bp), G6PD A− (968) Betica, Selma, Guantanamo (376A → G, 103 bp; 968T → C, 463 bp), G6PD Mediterranean, Dallas, Panama, “Sassari” (563C → T, 220 bp). Each PCR reaction was confirmed by an internal control (947pb).

For further confirmation of results, a set of 8 random samples with G6PD A+ (n = 4) and G6PD A− (n = 4) genotypes was sequenced. The PCR products were purified and sequenced at the Macrogen facilities (macrogenusa.com) using both forward and reverse primers. Chromas Pro and Mega5 software were used for sequence analysis. Positions 202 and 376 of exons IV and V were evaluated searching for the confirmatory SNP.

### Data analysis

Data were entered and verified using Microsoft Excel software^®^ and exported to SPSS version 21.0 (IBM, Armonk, New York, USA) for statistical analysis. Frequency distributions and percentages were calculated for sex, malaria infecting species and G6PD variants. The measure of agreement between PCR–RFLP and DiaPlexCTM G6PD Genotyping Kit (SolGent, Co., Ltd.) analysis was assessed by the Cohen’s kappa coefficient. A *p* value <0.05 was considered statistically significant. Strengthening the reporting of observational studies in epidemiology (STROBE) guidelines were followed whenever appropriate in reporting this study (see Additional file 1, STROBE statement) [39].

## Results

Of the initial 400 samples, 398 were successfully analyzed by PCR–RFLP, while the remaining two were excluded due to DNA amplification problems. Through genetic analysis it was determined that of the 398 samples, 214 (53.77%) and 184 (46.23%) were from female and male individuals, respectively. The frequency of two G6PDd allelic variants (A+ and A−) was determined in all samples. Overall 16% (64/398) of the samples analyzed had a G6PD A genotype. The distribution of the frequencies of G6PD A genotypes among the participants are shown in Tables 4 and 5.

**Table 4:** G6PD genotypes according to sex and *Plasmodium* species

Genotype	n	Sex	Total	*P. vivax*	*P. falciparum*	Mixed infection
			n (%)	n (%)	n (%)	n (%)
G6PD B (wildtype)	334	M	164 (41.21)	139 (34.92)	21 (5.28)	4 (1.01)
		F	170 (42.71)	145 (36.43)	22 (5.53)	3 (0.75)
G6PD A+	17	M	4 (1.01)	2 (0.50)	2 (0.50)	0
		F	13 (3.27)	10 (2.51)	3 (0.75)	0
G6PD A	47	M	16 (4.02)	8 (2.01)	7 (1.76)	1 (0.25)
		F	31 (7.79)	18 (4.52)	11 (2.76)	2 (0.50)
			398 (100)	322 (80.90	66 (16.58)	10 (0.02)

**Table 5:** Number of G6PD alleles among malaria-infected individuals according to sex

	n (%)	Genotype		
		G6PDA−	G6PDA+	G6PD B (wildtype)
Male	184 (46.23)	16 (4.02)	4 (1.01)	164 (41.21)
Female	214 (53.77)	31 (7.79)	13 (3.27)	170 (42.71)
Total	398 (100)	47 (11.81)	17 (4.27)	334 (83.92)

All 398 samples were genotyped by PCR–RFLP for the G6PDd A− allele (with two mutations in positions 376 A → G and 202 G → A). Sixteen (4.02%) were A− males, and 31 (7.79%) were A− females. For the G6PDd A+ allele (376A → G mutation), 4 (1.01%) were A+ males and 13 (3.27%) were A+ females (Table 5). The remaining 170 (42.71%) females and 164 (41.21%) males did not carry the aforementioned G6PDd alleles.

**Fig. 2:**
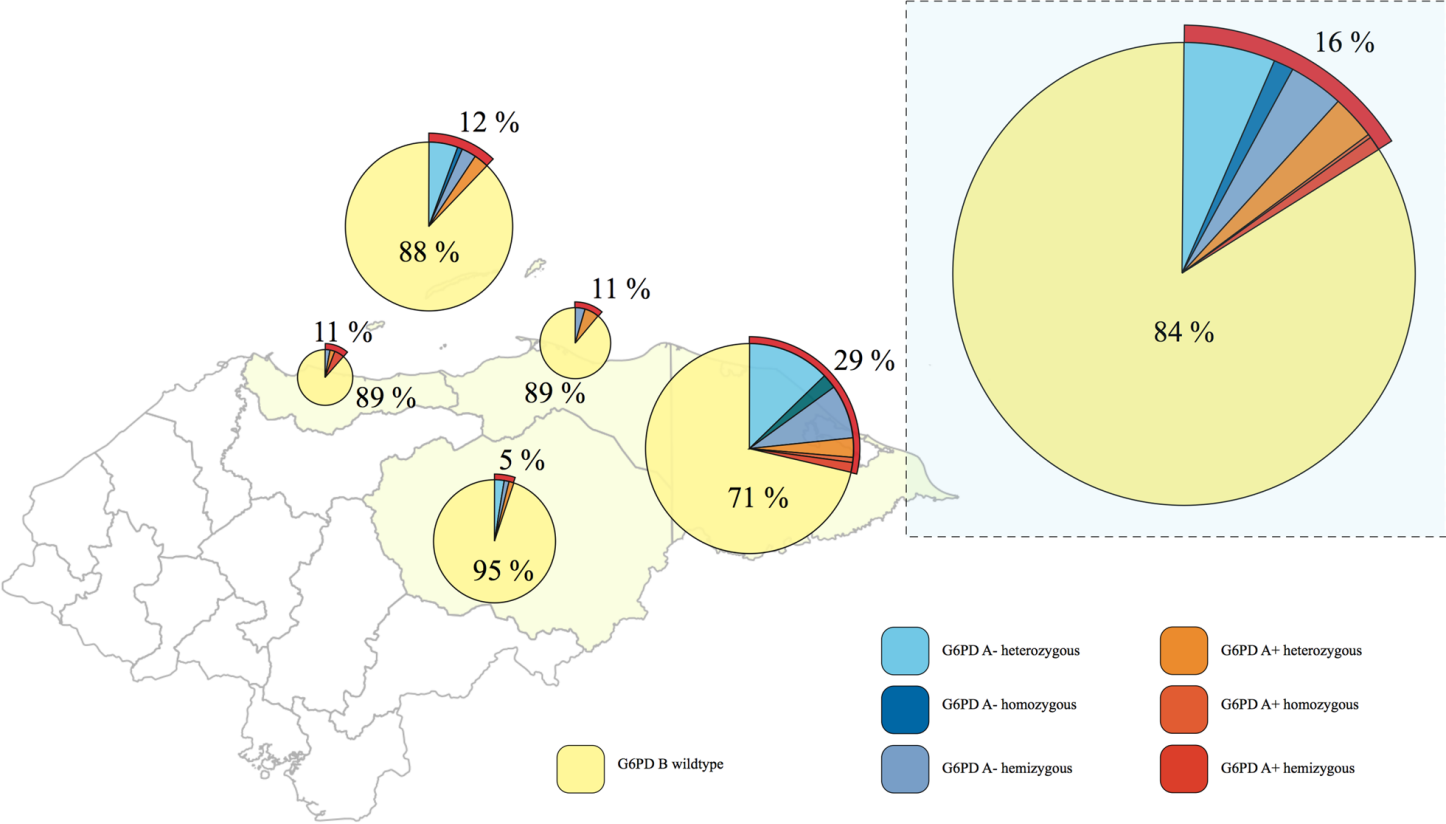
Distribution of G6PD variants from malaria-infected individuals.

Of the 398 individuals genotyped by PCR–RFLP, 17 (4.27%) showed the G6PDd A+ allele, of which 4 (23.53%) were A+ hemizygous males, 1 (5.88%) was an A+ homozygous female, and 12 (70.8%) were A+ heterozygous females. In addition, 47 of 398 (11.81%) samples exhibited the G6PD A− allele, of which 16 (34.04%) were A− hemizygous males, 6 (12.76%) were A− homozygous females, and 25 (53.1%) were A− heterozygous females. The remaining 334 (83.92%) individuals, −170 (42.71%) females and 164 (41.21%) males—did not carry either mutation (Fig. 2; Table 6).

**Table 6:** G6PD variants by geographical origin

Department	n	G6PD genotype	n (%)	Heterozygous	Homozygous	Hemizygous
Atlántida	35	A−	1 (2.86)	0	0	1
		A+	3 (8.57)	1	0	2
		B	31 (88.57)			
Colón	45	A−	2 (4.44)	0	2	0
		A+	3 (6.67)	3	0	0
		B	40 (88.89)			
Gracias a Dios	133	A−	31 (23.3)	17	3	11
		A+	7 (5.3)	4	1	2
		B	95 (71.4)			
Islas de la Bahía	107	A−	10 (9.35)	6	1	3
		A+	3 (2.80)	3	0	0
		B	94 (87.85)			
Olancho	78	A−	3 (3.85)	2	0	1
		A+	1 (1.28)	1	0	0
		B	74 (94.87)			
Total	398	A−	47 (11.81)	25	6	16
		A+	17 (4.27)	12	1	4
		B	334 (83.92)			

According to these figures, a male: female ratio of mutation prevalence was calculated at 1:1.8 (i.e., hemizygous males/(all homozygous females) + (10% of all heterozygous females) = 16/6 + (25 × 0.1%) = 1:1.88), based on previous studies describing this calculation and reviewed by Howes et al. [7].

As shown in Table 6, a higher proportion of individuals with the G6PDd A− genotype were from the departments of Gracias a Dios (31/133) and Islas de la Bahía (10/107).

To confirm the results obtained by PCR–RFLP, all 64 samples showing a G6PD A+ or G6PD A− genotype were analyzed using the commercial kit DiaPlexC™ G6PD Genotyping Kit (African type). The kit enables the detection of six variants of the G6PD gene, including those revealed through PCR–RFLP. Concordant results were obtained in all 47 samples with an A− genotype and 15 of the 17 samples with A+ genotype by PCR–RFLP. The discordant results revealed that one sample had an A− genotype whereas the other was a Santamaria mutation (376 A → G/542 A → T) (Table 7). In light of the absence of prior reports, this is likely the first time the Santamaria mutation has been identified in Honduras. According to these results, the measure of agreement between PCR–RFLP and the commercial Kit revealed a Cohen’s kappa coefficient = 0.923 (Std. error = 0.052). However, since the RFLP assay was not able to detect the 542 A → T mutation, the agreement between both approaches increased (*k* = 0.959).

**Table 7:** Comparison of two molecular techniques for the detection of G6PD variants

Solgent kit	PCR–RFLP		
	G6PD A−	G6PD A+	Total
G6PD A−	47 (72.3%)	1 (1.5%)	48 (73.8%)
G6PD A+	0	16 (24.6%)	16 (24.6%)
G6PD Santamaria	0	1 (1.5%)	1 (1.5%)
Total	47 (72.3%)	18 (27.7%)	65 (100%)

Measure of agreement Kappa = 0.923 with asymptotic Std. Error = 0.052 (not assuming the null hypothesis).

Of the eight samples with G6PD A+ (n = 4) and A− (n = 4) restriction patterns selected randomly for sequence analysis, all eight had the expected mutation in position 376. Conversely, the A− genotype could not be demonstrated in any of the four G6PD A− samples because of insufficient quality in the obtained sequences. A figure summarizing the overall study findings can be found as an additional file (see Additional file 2, Flowchart with overall study findings).

## Discussion

This study presents for the first time, data on the frequency of G6PDd genotypes in Honduras. For the analyzed samples, the results showed a frequency of G6PD A deficient genotypes of 16.08%, and a frequency of 11.81% with the A− genotype. As this study only aimed to detecting two of the most common G6PDd variants reported for the Latin American subcontinent, the existence of less frequent mutations (e.g., Mediterranean B−) cannot be ruled out.

These findings are consistent with a recent review of the literature from Latin America and the Caribbean, which concluded that the predominant genetic variant was A− (81.1% of the surveyed population) [25]; the same variant described in this study with 47 out of 66 samples showing a mutation (71.21%). According to this review, five countries have documented low prevalence rates of G6PDd (Argentina, Bolivia, Mexico, Peru, and Uruguay) whereas a prevalence >10% has been reported in four Caribbean islands, and some areas of Brazil, Colombia, and Cuba [25].

In the present study, the overall frequency of the deficient “African” genotype A− is greater than previous reports from highly prevalent areas in the Latin American subcontinent. These findings suggests that, as observed in other tropical regions of Latin America, G6PD deficiency is a common condition in Honduras, especially in areas that are inhabited by peoples of African descent.

In terms of the distribution of G6DPd by sex, the findings of the present study are in agreement with current global estimates. Recently, Howes et al. [7], applied a Bayesian geostatistical model to predict the allelic frequency in malaria endemic countries of class II and III G6PDd in both hemizygous men and homozygous or heterozygous women. They estimated that the frequency in hemizygous male population of Honduras could range from 100,000 to 500,000 (1.2–6% of the population). This projection holds true for the present study, as 4% of samples from male patients were hemizygous A−.

In terms of the proportion of hemizygous males compared with deficient females (either homozygous or heterozygous), both Howes et al. [7] and Monteiro et al. [25] coincide in a global estimate of 1:1.7. This estimate is also in agreement with the findings of the present study in which a 1:1.8 male:female ratio was found for deficient individuals.

Unfortunately, the lack of published data from most Central American countries prevents us from establishing sub-regional comparisons. A single publication from Panama reports the analysis of 75 children with G6PDd detected by neonatal screening. Eighty percent of these children had an A−^202A/376G^ genotype, while the remaining 20% had A−^376 G/968C^ or Mediterranean B− genotypes [26]. Another study from Costa Rica analyzed 289 Afro descendants from Port Limon, of which 28 (9.69%) had the “African” genotype A−^202A^ [29]. Altogether these results suggest that G6PD A− genotype might be the most common in Honduras, Panama and Costa Rica.

The distribution of G6PDd variants in malaria endemic areas of Honduras shows two departments with higher allele frequencies: Gracias a Dios (38/133, 28.6%) and Islas de la Bahía (13/107, 12.2%). In contrast with the rest of the country, where the population is predominantly Mestizo, Islas de la Bahía has a higher proportion of Afro descendant people (the English-speaking and Garifuna communities); whereas Gracias a Dios is largely inhabited by the Miskitos, an ethnic group with distant African Ancestry but nowadays considered Amerindian, an indigenous community [2, 40]. These data are consistent with the geostatistical model-based map proposed by Howes et al. [7] for African countries. They estimated that up to 37.5% of the sub-Saharan African territories would present an average prevalence of 10% while some countries would have a much higher prevalence, for instance Nigeria, where G6PDd prevalence could reach up to 31% [7]. In Honduras, it is believed that the ancestors of Afro descendant (Garifuna) populations originally came from Nigeria, which could explain the higher prevalence of G6PDd in some geographic areas of the country.

Without information on the ethnicity of the study samples, explaining the high proportion of the A− genotype found is difficult. However, based on current population demographics originating from colonization and post-colonization periods, it could be expected that a countrywide G6PDd survey would confirm higher prevalence of the “African” genotype in the same departments.

The high frequency of the G6PD A− variant among malaria patients in Honduras lends support to the well-known hypothesis correlating their geographical distribution [41–43]. Several studies suggest that the G6PDd provides protection against *Plasmodium* spp. infection in non-immune adults [42, 44, 45]. Further, population genetic analyses of the G6PD locus indicate that these mutations have arisen recently in certain geographical areas as a result of positive selection exerted by malaria [46–49]. Based on these observations, it could be predicted that the prevalence of G6PDd variants would be higher in the Atlantic coast of Honduras where the incidence of malaria is higher relative to the rest of the country [50]. Naturally, additional research utilizing a more representative sample of the Honduran population is warranted to confirm our findings.

Future prevalence studies of the general population should also endeavor to include samples from both malaria-negative individuals and individuals residing in non-endemic areas of the country. Lacking these reference groups is an important limitation of the present study. Notwithstanding, our results suggest that a significant sector of the Honduran population might potentially be at risk of adverse effects due to PQ treatment.

The use of 8-aminoquinolines in individuals with G6PDd has the potential to trigger adverse clinical effects, and therefore it would not be reasonable nor responsible to give one of these drugs before analyzing the patient’s G6PD status [51]. However, the severity of side effects of 8-aminoquinolines varies considerably depending on the deficient G6PD phenotype [52] as well as the dose of the drug [53, 54].

Four percent of the individuals analysed here were G6PDd A− hemizygous males while 1% of them were homozygous females. These individuals could be potentially harmed by the administration of PQ, the only drug available in Honduras to eliminate liver *P. vivax* hypnozoites (and thus prevent relapses). In terms of PQ, the Honduran national regulation for malaria control establishes that treatment should consist of 0.25 mg/kg/day for 14 days to patients with uncomplicated vivax malaria, and 0.75 mg/kg as single dose the first day of treatment for a *P. falciparum* infection [4]. This regulation follows international recommendations and is considered safe for individuals with G6PDd [55]. This treatment scheme seems to trigger fewer adverse effects than others of shorter duration but with higher PQ doses, as described in the National regulation of Honduras’s neighbouring countries.

Based on the importance that malaria elimination programs have acquired [56], Monteiro et al. have recently published a systematic review concerning the clinical complications of G6PDd in Latin America [54]. The review revealed that although the number of complications reported after PQ intake was low—probably due to misreporting or inappropriate diagnosis—haemolysis was the most common complication among PQ-treated G6PDd-individuals in Latin America.

While the number of complicated cases triggered by PQ could be low, the risk of haemolysis in individuals with a class II or III deficiency might override the benefits of malaria treatment. In turn, failure to treat malaria may put the patient at risk of serious clinical disease [17]. Hence, prior determination of G6PD status is necessary before the prescription of 8-aminoquinoline drugs, such as PQ, or more recently tafenoquine (TQ), a drug in its final phase of development as a promising anti-malarial agent [57–59].

The diagnosis and management of G6PDd is currently limited by cost, infrastructure and logistics. Fast, low-cost, and easy techniques are urgently needed for malaria-endemic countries such as Honduras. This is particularly true for remote, high-transmission areas where disadvantaged populations of African descent reside, as they might be more at risk of developing PQ-triggered haemolytic events [60, 61]. National policies establishing malaria management with oxidizing drugs such as PQ or TQ ought to be informed by research evidence concerning the frequency of local G6PDd variants.

## Conclusions

This study documented for the first time the frequencies of the G6PDd A+ and A− genotypes through two genotypic approaches among malaria-infected individuals in Honduras. These frequencies were higher than the average expected for Latin America. Although ethnic groups potentially carrying genetic mutations in the G6PDd gene might have been overrepresented in this study, these populations reside in areas where malaria transmission is the highest in the country. Therefore, this report highlights the need for immediate research to determine whether these populations are at risk of PQ-triggered haemolytic complications. Additionally, our findings contribute to filling the knowledge gaps regarding the distribution and diversity of G6PD variants in Latin America.

## Additional files

Additional file 1: STROBE statement. Additional file 2: Flowchart with overall study findings.

## Abbreviations

G6PD: glucose-6-phosphate dehydrogenase
G6PDd: glucose-6-phosphate dehydrogenase deficiency/deficient
NADPH: nicotinamide adenine dinucleotide phosphate reduced
PQ: primaquine
TQ: tafenoquine
WHO: World Health Organization
UNAH: National Autonomous University of Honduras
PCR: polymerase chain reaction
18S rRNA: 18S ribosomal RNA
RFLP: restriction fragment length polymorphism
OMIM: online Mendelian inheritance in man
SNP: single nucleotide polymorphism
