# Supplementary figures and images for: Glucose-6-phosphate dehydrogenase deficiency among malaria patients of Honduras: a descriptive study of archival blood samples
Source: Malar J. 2015 Aug 7;14:308. doi: 10.1186/s12936-015-0823-z (PMC4528855; doi:10.1186/s12936-015-0823-z)

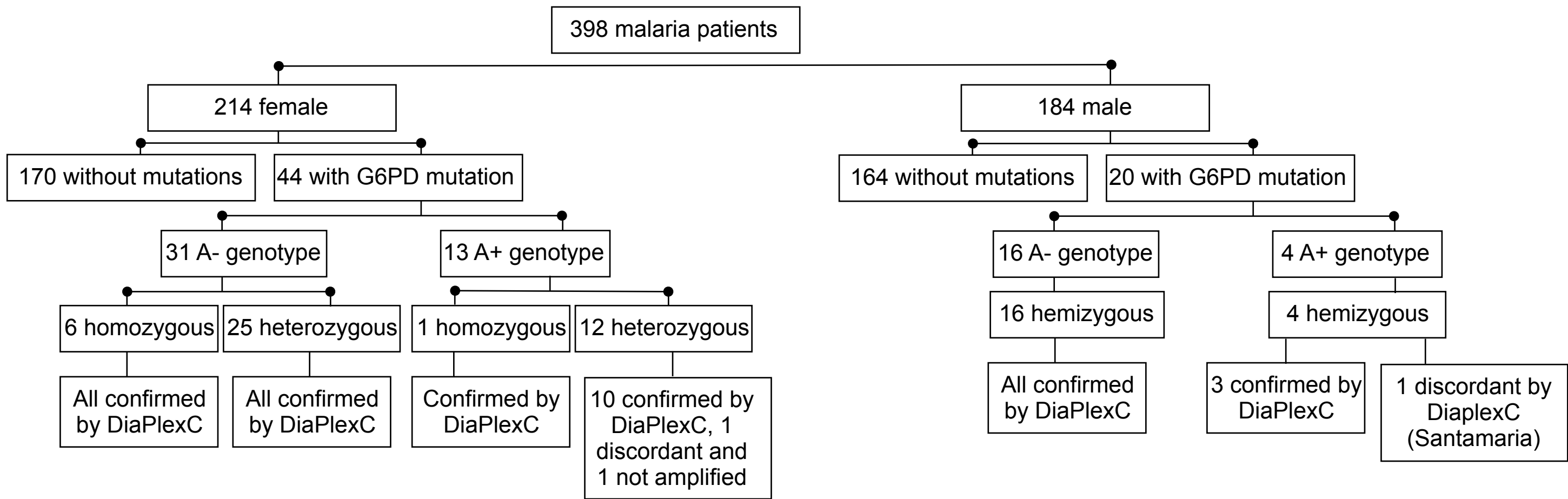

Supplement: Additional file 2: — Flowchart with overall study findings. [file 12936_2015_823_MOESM2_ESM.pdf]
